# Hospitalization Frequency and Charges for Neurocysticercosis, United States, 2003–2012

**DOI:** 10.3201/eid2106.141324

**Published:** 2015-06

**Authors:** Seth E. O’Neal, Robert H. Flecker

**Affiliations:** Oregon Health & Science University, Portland, Oregon, USA

**Keywords:** Neurocysticercosis, cysticercosis, neglected tropical disease, economic burden, *Taenia solium*, epilepsy, brain disease, parasitic disease, helminthiasis, cestode infection, central nervous system diseases, parasites

## Abstract

Neurocysticercosis, brain infection with *Taenia solium* larval cysts, causes substantial neurologic illness around the world. To assess the effect of neurocysticercosis in the United States, we reviewed hospitalization discharge data in the Nationwide Inpatient Sample for 2003–2012 and found an estimated 18,584 hospitalizations for neurocysticercosis and associated hospital charges totaling >US $908 million. The risk for hospitalization was highest among Hispanics (2.5/100,000 population), a rate 35 times higher than that for the non-Hispanic white population. Nearly three-quarters of all hospitalized patients with neurocysticercosis were Hispanic. Male sex and age 20–44 years also incurred increased risk. In addition, hospitalizations and associated charges related to cysticercosis far exceeded those for malaria and were greater than for those for all other neglected tropical diseases combined. Neurocysticercosis is an increasing public health concern in the United States, especially among Hispanics, and costs the US health care system a substantial amount of money.

## Abstract 2

Total hospital charges exceeded US $900 million, and nearly three-quarters of hospitalized patients were Hispanic.

Neurocysticercosis is a leading cause of acquired epilepsy in the developing world (*1**,**2*). The disease occurs when larvae of the pork tapeworm, *Taenia solium*, encyst in the human brain; this process causes a broad range of neurologic signs and symptoms, including seizures, headache, obstructive hydrocephalus, encephalitis, stroke, and cognitive and other mental health disorders (*3**,**4*). Neurocysticercosis is endemic in poor rural communities in Latin America, sub-Saharan Africa, and Asia, where pigs can access and ingest human feces (Figure 1). However, the disease is also of increasing public health concern in the United States, especially in the immigrant population and among persons who have traveled to regions where cysticercosis is endemic (*5*).

**Figure 1:**
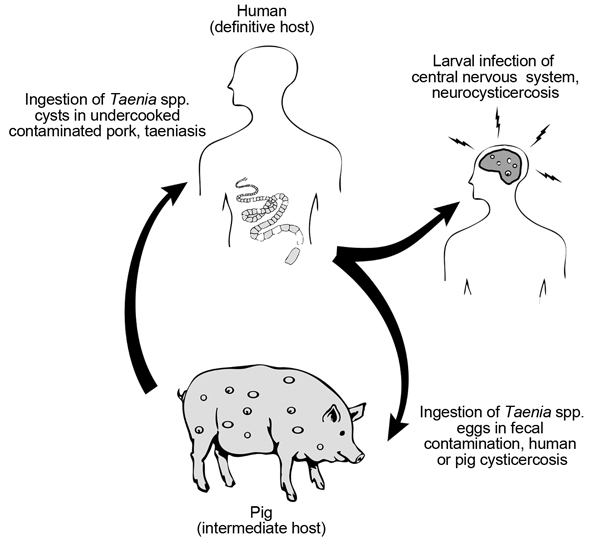
The lifecycle of the *Taenia solium* cestode parasite.

The World Health Organization designates cysticercosis as a neglected tropical disease (NTD) and has called for international efforts to strengthen surveillance (*6**–**8*). The disease remains neglected partly because the scale of the problem has not been well defined (*2*). In most disease-endemic regions, population-level data are sparse because surveillance for neurocysticercosis is nonexistent and diagnostic neuroimaging is typically unavailable. In the United States, there is an opportunity to collect population-based data on neurocysticercosis because of the large immigrant population at risk for infection, the widespread availability of neuroimaging, and the well-established disease surveillance infrastructure. However, only Alaska, Arizona, California, New Mexico, Oregon, and Texas require reporting of neurocysticercosis.

Death rates due to neurocysticercosis in the United States have been reported previously (*9*), but national-level assessments of neurocysticercosis that use population–based data are lacking. The objective of our study was to evaluate the frequency and total associated charges for hospitalizations due to neurocysticercosis in the United States and to compare these against other tropical diseases of potential importance in the United States.

## Methods

### Data Source

We analyzed hospital discharge data contained in the Nationwide Inpatient Sample (NIS) for 2003–2012 (*10**,**11*). The NIS, a stratified weighted sample of short-term and nonfederal hospitals, is designed to approximate a 20% sample of all community hospitals in the United States. As of 2012, 47 states participated in reporting discharge data to the NIS (only Alabama, Delaware, Idaho, and the District of Columbia had not participated), creating a sample representing 95% of the national population. The NIS is the largest collection of longitudinal inpatient care data in the United States and holds information on ≈8 million hospitalizations from >1,000 hospitals each year (*10*). NIS data are de-identified and include information on demographics, diagnostic and procedural codes, length of stay, discharge status, total charges, and expected payees associated with each hospitalization.

### Case Definitions

We based our case definitions for hospitalization on diagnostic and procedural codes from the International Classification of Diseases, 9th Revision, Clinical Modification (ICD-9-CM). The ICD-9-CM code listed in the first diagnostic field is intended to capture the primary reason for hospitalization. However, there is no specific ICD-9-CM code for neurocysticercosis, so coding patterns may vary. For example, a hospitalization for neurocysticercosis might be coded with a first diagnostic field of 123.1 (cysticercosis) or with a neurologic code, such as 345.9 (epilepsy unspecified), in combination with 123.1 in a different diagnostic field.

We used 2 case definitions in this analysis. The first was a conservative case definition for reporting hospitalizations associated with neurocysticercosis. This definition required the ICD-9-CM code for cysticercosis (123.1) in any of the 15 available diagnostic fields and a supporting diagnostic or procedural code associated with a clinical manifestation of neurologic disease in any of the first 5 diagnostic or procedural fields (Table 1). We used individual ICD-9-CM codes and coding groups defined by Clinical Classification Software to define these additional diagnostic or procedural codes (*13*). This conservative case definition was designed to reduce the likelihood of including hospitalizations for persons carrying an existing diagnosis of neurocysticercosis who were hospitalized for an unrelated condition.

**Table 1:** Supporting diagnostic or procedural codes from ICD-9-CM used for conservative case definition for reporting hospitalizations associated with neurocysticercosis*

CCS code	Diagnosis or procedure
Diagnostic code	
76	Meningitis
77	Encephalitis
78	Other CNS infection and poliomyelitis
83	Epilepsy; convulsions
84	Headache; including migraine
85	Coma; stupor; and brain damage
90	Inflammation; infection of eye
95	Other nervous system disorders
109	Acute cerebrovascular disease
111	Other and ill-defined cerebrovascular disease
112	Transient cerebral ischemia
245	Syncope
650	Adjustment disorders
651	Anxiety disorders
652	Attention-deficit, conduct, and disruptive behavior disorders
653	Delirium, dementia, and amnestic and other cognitive disorders
656	Impulse control disorders, NEC
657	Mood disorders
658	Personality disorders
659	Schizophrenia and other psychotic disorders
662	Suicide and intentional self-inflicted injury
670	Miscellaneous mental disorders
Procedural codes	
1	Incision and excision of CNS
2	Insertion; replacement; or removal of extracranial ventricular shunt
177	Computerized axial tomography (CT) scan head
198	Magnetic resonance imaging
199	Electroencephalogram (EEG)

*A complete list of ICD-9-CM codes used in this study is provided in the online Technical Appendix (http://wwwnc.cdc.gov/EID/article/21/6/14-1324-Techapp1.pdf). ICD-9-CM, International Classification of Diseases, 9th Revision, Clinical Modification; CCS, Clinical Classification Software groupings of ICD-9-CM codes (*12*); CNS, central nervous system; NEC, not elsewhere classified.

The second case definition was designed to facilitate comparison of hospitalizations for cysticercosis with those for the 16 other NTDs and malaria. The case definition for cysticercosis included all hospitalizations with an ICD-9-CM diagnostic code for cysticercosis (123.1) in any of the first 15 diagnosis fields, but it did not require an additional supportive diagnostic or procedural code. Similarly, the case definitions for the other tropical diseases in the comparative analysis relied on ICD-9-CM codes specific to the disease without a requirement for an additional supportive diagnostic or procedural code. This approach ensured consistency of case definitions across the various diseases at the expense of greater specificity. We assumed that the likelihood of capturing unrelated hospitalizations was similar for the diseases we compared. We excluded Buruli ulcer from our comparison because there is no ICD-9-CM code specific for this disease. However, to our knowledge, Buruli ulcer has not been reported in the United States (*14*). We did not report hospitalizations for rabies, African trypanomiasis, or dracunculiasis because the numbers of hospitalizations were too low (<10/year) to provide accurate estimates. A list of ICD-9-CM codes used in all case definitions is provided in the Technical Appendix.

### Statistical Methods

To account for the sampling design of the NIS, we analyzed all data by using the survey family of commands in Stata 13 (StataCorp LP, College Station, TX, USA). We applied hospital discharge weights provided in the NIS to estimate total national hospitalizations on the basis of the stratified sample. All sampled hospitals, regardless of whether they had a patient who was hospitalized with neurocysticercosis, were included for calculation of SEs and CIs. We examined neurocysticercosis hospitalizations by patient age, sex, race, place of service, discharge status, and length of stay; US region; associated diagnostic and procedural codes; and hospitalization charges. State-level assessment was not possible because of the sampling and stratification strategy used in the NIS. Mean annual hospitalization rates were calculated as the weighted number of hospitalizations per 100,000 population on the basis of the US Census Bureau data for each year during the study period (*15*). Age- and sex-adjusted rates were calculated by using the direct standardization method and the 2005 US Census population as the reference population.

We used Gaussian family generalized linear models with logarithmic function link within the Stata survey framework to estimate the crude and adjusted mean length of stay and mean hospitalization charges. We first constructed univariate generalized linear models to evaluate demographic variables of interest, retaining those that were significant at the p<0.2 level (Wald test) in the final multivariate models. The independent categorical variables we evaluated were sex, age, race, hospital region, and year of hospitalization. Once we built the final models, we estimated the mean length of stay and mean hospitalization charges for diagnoses and procedures commonly seen with neurocysticercosis (i.e., seizures, obstructive hydrocephalus, headache, stroke, mental health disorder, encephalitis/meningitis, cerebral edema, syncope, neuroimaging, ventricular shunt management, and central nervous system surgery) by individually introducing dummy variables encoding these clinical variables into the models. Inflation-adjusted charges were used in all models.

### Hospital Charges

We analyzed hospital charges that were billed to private insurance, Medicaid, Medicare, and other sources from the payer’s perspective. Charges represent the amount that hospitals billed for services, not the actual cost of providing these services. Generally, total charges did not include professional fees, noncovered charges, or charges incurred in the emergency department unless the patient was admitted directly from the emergency department into the hospital. We adjusted all charges for inflation by using the Consumer Price Index, setting the base year to 2012.

## Results

During 2003–2012, an estimated 23,266 hospitalizations (95% CI 21,741–24,792) in the United States were assigned an ICD-9-CM code of 123.1 in any of the first 15 diagnostic fields. Of these hospitalizations, 18,584 (95% CI 17,322–19,846), approximately 80% of the total, met our case definition of hospitalization due to neurocysticercosis. The number of hospitalizations due to neurocysticercosis per year ranged from a high of 2,247 in 2006 to a low of 1,495 in 2012. The largest proportion of hospitalizations due to neurocysticercosis occurred in the western region (n = 8,026, 42.9% [95% CI 39.2%–46.7%)], followed by the southern region (n = 5,860, 31.8% [95% CI 28.6%–35.1%]), the northeastern region (n = 2,902, 15.5% 95% CI [13.5%–17.6%]) and the midwestern region (n = 1,796, 9.8% [95% CI 8.2%–11.7%]).

We found distinct differences in the mean annual incidence rates of hospitalization stratified by age, sex, and race (Table 2). The mean annual incidence of hospitalization was highest in 20- to 44-year-old age group (1.04 hospitalizations/100,000 population). Hospitalization rates were 33% higher among male patients than female patients. The age- and sex-adjusted mean annual incidence of hospitalizations was highest among Hispanics (2.50 hospitalizations/100,000 population); the rate was 35 times higher than that for non-Hispanic whites, 10 times higher than that for blacks, and 8 times higher than that for Asian/Pacific Islanders. Unadjusted rates by race were similar: Hispanic, 2.57/100,000; white, 0.06/100,000; black, 0.23/100,000; and Asian/Pacific Islander, 0.26/ 100,000.

**Table 2:** Number and rate of hospitalizations for neurocysticercosis in the United States, by demographic group, 2003–2012*

Characteristic†	No. hospitalizations (SE)	% All hospitalizations (95% CI)	Rate (95% CI)‡
Age, y			
<20	1,493 (103)	8.0 (7.1–9.1)	0.18 (0.16–0.21)
20–44	10,827 (394)	58.3 (56.5–60.1)	1.04 (0.97–1.12)
45–64	4,357 (232)	23.5 (22.0–25.0)	0.56 (0.51–0.62)
≥65	1,889 (136)	10.2 (9.0–11.5)	0.49 (0.42–0.56)
Sex			
M	10,377 (373)	56.3 (54.5–58.2)	0.70 (0.65–0.75)
F	8,043 (349)	43.7 (41.8–45.5)	0.52 (0.48–0.57)
Race/ethnicity			
Hispanic	12,030 (551)	74.0 (71.5–76.3)	2.50 (2.27–2.73)
White	1,530 (104)	9.4 (8.2–10.7)	0.07 (0.06–0.08)
Black	900 (95)	5.5 (4.5–6.8)	0.25 (0.21–0.30)
Asian/Pacific Islander	377 (61)	2.3 (1.7–3.2)	0.31 (0.23–0.39)
Overall	18,584 (644)		0.61 (0.57–0.66)

*National estimates were based on the Nationwide Inpatient Sample, by using diagnosis code 123.1 from the International Classification of Diseases, 9th  Revision, Clinical Modification. †Missing data not presented ‡Rate for age and sex are unadjusted. Rate for race/ethnicity is adjusted for age and sex by direct standardization method by using 2005 US Census data. Rates expressed as mean annual incidence per 100,000 population.

### Length of Stay, Total Charges, and Payees

The mean length of hospitalization was 6.0 (95% CI 5.7–6.4) days and did not show a significant trend over the study period (p = 1.0). Total inflation-adjusted hospitalization charges over the 10-year study period were US $908,238,000 (95% CI US $814,483,000–$1,001,992,000), increasing 27% from US $72,560,000 in 2003 to US $91,959,000 in 2012. The mean charge per hospitalization was US $50,976 (95% CI US $47,492–$54,716), increasing 50% over the 10-year study period from US $41,874 in 2003 to US $62,986 in 2012. After we adjusted for demographic variables, mean length of stay and mean hospitalization charges were substantially higher for male patients, middle-aged adult patients, and patients from the western region (online Technical Appendix). Publically funded insurance (Medicaid or Medicare) was the primary payer in 40% of the hospitalizations (Table 3).

**Table 3:** Source of admission, disposition, and expected payer for hospitalizations due to neurocysticercosis, United States, 2003–2012*

Characteristic	No. hospitalizations (SE)	% All hospitalizations (95% CI)
Source of admission		
Emergency department	9,436 (484)	74.7 (72.3–76.9)
Routine	2,210 (145)	17.5 (15.6–19.6)
Transfer	947 (80)	7.5 (6.4–8.7)
Disposition		
Routine	15,693 (562)	84.5 (83.1–85.8)
Transfer	1,617 (107)	8.7 (7.7–9.8)
Home health	834 (81)	4.5 (3.8–5.4)
Against medical advice	205 (33)	1.1 (0.8–1.5)
Died	218 (32)	1.2 (0.9–1.6)
Expected primary payer		
Medicare	2,025 (139)	10.9 (9.7–12.3)
Medicaid	5,543 (316)	29.9 (27.8–32.1)
Private insurance	4,335 (206)	23.4 (21.4–25.4)
Self-pay	4,753 (224)	25.6 (23.8–27.5)
Other payer	1,883 (160)	10.2 (8.9–11.6)
*National estimates were determined on the basis of the Nationwide Inpatient Sample, by using diagnosis code 123.1 from the International Classification of Diseases, 9th Revision, Clinical Modification.		

### Associated Diagnoses and Procedures

The most common diagnosis group associated with hospitalization for neurocysticercosis was epilepsy/convulsions, which occurred in 57.3% of hospitalizations, followed by obstructive hydrocephalus (17.7%) and headache (12.4%) (Table 4). After we controlled for year and patient demographics, the diagnoses associated with the longest mean length of stay and the highest mean charges were encephalitis/meningitis (12.2 days and US $78,984) and hydrocephalus (11.4 days and US $79,084). Diagnostic codes for syncope and headache were associated with the shortest stays and lowest charges (3.4 days and US $20,017 and 3.8 days and US $19,893, respectively). Procedure codes for shunt management (insertion, removal, or repair) were associated with a mean length of stay of 16.3 days and mean hospitalization charges of US $86,272; codes for brain surgery (central nervous system incision or excision) were associated with a mean length of stay of 10.3 days and mean hospitalization charges of US $89,893. Only 17% of hospitalizations included a procedural code for either computed tomography scans or magnetic resonance imaging of the head.

**Table 4:** Diagnostic and procedure codes for hospitalizations due to neurocysticercosis, United States, 2003–2012*

Associated diagnoses and procedures	No. hospitalizations (SE)	% All hospitalizations (95% CI)	Mean length of stay, d†	Mean charges, US$†
Diagnoses				
Epilepsy; convulsions	10,652 (360)	57.3 (55.3–59.3)	5.4 (4.2–6.9)	33,058 (21,846–50,023)
Obstructive hydrocephalus	3,292 (208)	17.7 (16.2–19.3)	11.4 (8.1–16.2)	79,084 (46,139–135,552)
Headache, including migraine	2,308 (126)	12.4 (11.3–13.6)	3.8 (2..9–4.9)	19,893 (12,422–31,857)
Cerebrovascular disease	1,650 (121)	8.9 (7.9–10.0)	7.5 (5.3–10.6)	45,183 (26,027–78,436)
Mental health disorder	1,843 (132)	9.9 (8.8–11.2)	6.4 (4.7–8.8)	24,436 (13,578–43,979)
Encephalitis/meningitis	1,033 (81)	5.6 (4.8–6.4)	12.2 (8.1–18.6)	78,983 (46,851–133,151)
Cerebral edema	931 (79)	5.0 (4.3–5.9)	7.5 (5.6–10.0)	40,639 (23,449–70,429)
Syncope	573 (56)	3.1 (2.6–3.7)	3.4 (2.6–4.6)	20,017 (11,934–33,577)
Procedures				
Neuroimaging, CT of head or MRI	3,087 (330)	16.6 (13.9–19.8)	6.1 (4.8–7.7)	34,905 (22,177–54,937)
Ventricular shunt, insert, remove, or repair	1,661 (137)	8.9 (7.9–10.2)	16.3 (10.6–25.1)	86,272 (48,313–154,054)
CNS incision or excision	1,499 (111)	8.1 (7.1–9.2)	10.3 (7.9–13.5)	89,893 (56,625–142,709)

*National estimates were based on the Nationwide Inpatient Sample, by using diagnosis code 123.1 from the International Classification of Diseases, 9th Revision, Clinical Modification. CNS, central nervous system; CT, computed tomography; MRI, magnetic resonance imaging. †Mean length of stay and mean inflation-adjusted hospitalization charges for diagnostic and procedure codes after adjusting for year, patient age, sex, race, and hospital region. Diagnostic and procedure codes were evaluated individually as independent variables in the final generalized linear models built for length of stay and charges.

### Comparison of NIS Data for Cysticercosis with that for NTDs and Malaria

**Figure 2:**
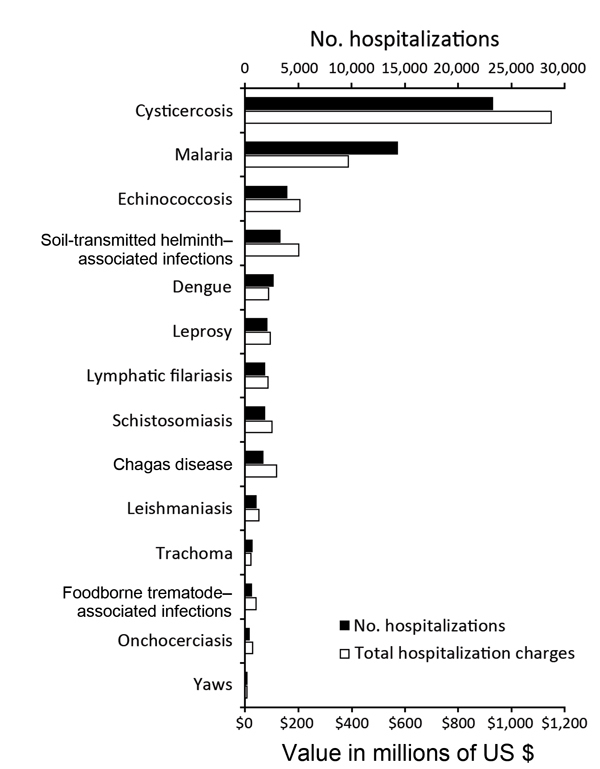
Frequency and total charges of hospitalizations in the United States during 2003–2012 for 13 of the World Health Organization (WHO)–designated neglected tropical diseases (NTDs) and malaria. Estimates were determined by using the Nationwide Inpatient Sample, which codes diagnoses according to the International Classification of Diseases, 9th Revision, Clinical Modification. Frequency of and total charges for hospitalizations for the other NTDs (i.e., Buruli ulcer, rabies, African trypanomiasis, and dracunculiasis) are not shown because there were too few hospitalizations for these diseases for accurate estimation. Frequency and total charges of hospitalizations for malaria, although it is not one of the WHO–designated NTDs, are shown for comparison.

The frequency of and total charges for hospitalizations due to cysticercosis exceeded those for all other NTDs combined (Figure 2). During 2003–2012, an estimated 23,266 (95% CI 21,741–24,792) hospitalizations were associated with a diagnosis code for cysticercosis, resulting in US $1,149,044,000 in total hospital charges (95% CI US $1,038,730,000–$1,259,357,000). In contrast, there were 20,029 hospitalizations and US $1,043,109,000 in total charges for all of the other NTDs combined (Table 5).

**Table 5:** Hospitalizations and total charges for neglected tropical diseases and malaria, United States, 2003–2012*

Disease	Hospitalizations			Total charges	
	No. (SE)	95% CI		US$, millions (SE)	95% CI
Cysticercosis	23,266 (778)	21,741–24,792		1,149 (56)	1,039–1,259
Malaria	14,319 (434)	13,469–15,169		387 (18)	351–423
Echinococcosis	3,919 (170)	3,586–4,252		206 (16)	174–237
Soil-transmitted helminth–associated infections	3,256 (151)	2,959–3,552		201 (19)	162–239
Dengue	2,644 (135)	2,379–2,909		89 (9)	70–107
Leprosy	2,055 (135)	1,791–2,319		94 (9)	76–111
Lymphatic filariasis	1,836 (106)	1,629–2,044		86 (9)	68–103
Schistosomiasis	1,811 (120)	1,576–2,046		101 (12)	78–125
Chagas disease	1,686 (151)	1,389–1,982		118 (17)	84–152
Leishmaniasis	1,022 (92)	841–1,203		52 (7)	38–66
Trachoma	649 (69)	514–784		20 (4)	13–28
Foodborne trematode–associated infections	610 (60)	492–729		41 (7)	28–54
Onchocerciasis	380 (47)	287–473		29 (12)	5–53
Yaws	161 (28)	106–216		7 (2)	3–11

*National estimates were determined on the Nationwide Inpatient Sample by using diagnostic codes from the International Classification of Diseases, 9th Revision, Clinical Modification. A complete list of ICD-9-CM codes used in this study is provided in the online Technical Appendix (http://wwwnc.cdc.gov/EID/article/21/6/14-1324-Techapp1.pdf).

## Discussion

The study findings demonstrate that neurocysticercosis poses considerable health and economic problems in the United States, especially among the Hispanic population. Over the 10-year study period, >18,500 hospitalizations for neurocysticercosis occurred, totaling hospital charges of >US $908 million, of which 40% was billed to publicly funded insurance programs. Hospitalization stays were prolonged and expensive, reflecting the complicated nature of acute disease management. Hospitalizations and associated charges for cysticercosis exceeded the totals for malaria and for all of the other NTDs combined.

The hospitalization rates we report in this nationwide study are comparable to those reported in previous state- or county-level studies, providing support for the case definition we used (*12*,*15**–**20*). Because there is no ICD-9-CM diagnostic code specific for neurocysticercosis, the case definitions varied slightly among these studies. The estimated overall hospitalization rate of 0.65/100,000 population that we report falls between the rates previously observed in California (0.8–1.1 hospitalization/100,000 population) and Oregon (0.2–0.5 hospitalizations/100,000 population) (*12*,*15**–**18*). Risk for hospitalization was highest among Hispanic, male, and young to middle-aged adult patients in all studies.

Nearly three quarters of all patients hospitalized for neurocysticercosis in the United States were Hispanic. The Hispanic population is the largest minority group in the United States and among the fastest growing US population groups. Because the hospitalization rate for the Hispanic population is 36 times greater than that of the non-Hispanic white population, the effect of neurocysticercosis on the US economy is likely to increase substantially in the coming years. The US Census Bureau projects that the Hispanic population will grow from 53 million in 2012 to >78 million by 2030 (*21*). Without changes in the rate of hospitalization or the increase in mean hospitalization charges, there could be >1,900 hospitalizations and US $250 million total charges related to neurocysticercosis among Hispanics alone in the year 2030. Changing immigration patterns may also bring an influx of cases in persons from other regions of the world where neurocysticercosis is endemic, particularly Asia and sub-Saharan Africa.

Several hospital-based studies have shown that seizures are the most frequent reason for hospitalization for neurocysticercosis (*3**,**4**,**22*). In this study, epilepsy was the most frequent diagnosis associated with hospitalization for neurocysticercosis; it was coded in more than half of all hospitalizations for the disease. Seizures in neurocysticercosis are typically amenable to therapy with antiepileptic and anti-inflammatory drugs, resulting in relatively uncomplicated and short hospital stays. In contrast, more severe disease may require intensive interventions and longer hospitalizations, resulting in higher charges (*23**–**25*). While diagnoses of obstructive hydrocephalus or encephalitis/meningitis occurred in ≈20% of persons hospitalized for neurocysticercosis, these more severe presentations accounted for 40% of the total charges incurred.

We report hospitalization diagnostic codes that may not represent the distribution of disease manifestations experienced by individual patients. For example, although a diagnostic code for headache was listed for 11% of hospitalized patients, only patients with headaches associated with underlying pathology requiring acute intervention, such as obstructive hydrocephalus, are likely to be admitted and therefore represented in this study. Even then, the diagnosis of headache may be underrepresented. There were twice as many hospitalizations with diagnostic codes for hydrocephalus and encephalitis than for headache, although both of these manifestations would be expected to be associated with headache (*22*). Similar caution is suggested in interpreting the frequency of other diagnoses presented here. It may seem contradictory that only 17% of hospitalizations had a procedural code for neuroimaging. However, because most imaging for neurocysticercosis would be expected to occur in the emergency department before admission, the infrequent coding for neuroimaging may reflect exclusion of these procedural codes from the hospital discharge summary.

This study documents the substantial costs of hospitalizations due to neurocysticercosis in the United States, but the true effect of neurocysticercosis on the US health care system is likely much greater. Only those emergency department visits that result directly in inpatient admission are captured in the hospital discharge databases in the NIS. In Oregon (*15*), over 40% (31/72) of all patients with neurocysticercosis were seen only in the emergency department and were not admitted to the hospital. While nonadmissions likely represent cases of less clinical severity, substantial charges are still incurred in the emergency department and in outpatient follow-up. Neurocysticercosis is also likely to be substantially underdiagnosed and misdiagnosed because of the lack of a definitive diagnostic test and limited provider awareness of the disease.

Neurocysticercosis also often results in chronic disease that requires outpatient follow-up with infectious disease or neurology specialists, none of which is captured in this study. Management of neurocysticercosis may involve long-term antiepileptic therapy, prolonged regimens of antiparasitic drugs and high-dose corticosteroids, monitoring and repair of ventriculoperitoneal shunts, and treatment of frequent complications resulting from these interventions (*26**,**27*). A chart review at the outpatient neurology clinic in a Houston hospital showed that 2% of all patients were seen for management of neurocysticercosis (*28*). A few states are now collecting comprehensive claims data covering health care provided in inpatient, outpatient, and long-term care settings. Data from these programs could provide more complete information about health care and associated costs related to management of neurocysticercosis in all settings. The high neurocysticercosis hospitalization rate we noted in young adults and men suggests substantial indirect costs to the US domestic workforce. Loss of worker productivity should also be considered in the overall costs of neurocysticercosis.

The use of administrative databases, such as the NIS, to obtain data for this study does have drawbacks, including several limitations we already described. An additional drawback to using the NIS was the inability to identify multiple hospitalizations for a single person, which precludes the ability to estimate the prevalence or incidence of disease. Although the number of states participating in NIS has grown over the years, several states still do not participate in reporting hospital discharge data. In addition, the regional sampling structure of the NIS does not allow for accurate state-level estimates, limiting the ability to identify specific states whose populations are at increased risk for neurocysticercosis. Furthermore, the lack of in-depth demographic and clinical information in the NIS limits the type of questions that can be addressed. For example, knowing the country of birth or travel history of patients with neurocysticercosis could help understand their source of exposure.

Although the primary purpose of this study was to evaluate hospitalizations for neurocysticercosis, we also compared hospitalizations for cysticercosis with those for other NTDs and malaria. Our findings showed that the number of hospitalizations for cysticercosis was nearly 2 times the number for malaria, and the associated hospital charges were nearly 3 times higher. In addition, hospitalizations and charges for cysticercosis were higher than those for all other NTDs we evaluated combined. This comparative analysis was not meant to be exhaustive; we recognize that many factors other than hospitalization contribute to the public health effect of any particular disease. However, the markedly higher number of hospitalizations and associated charges related to cysticercosis, compared with those for other NTDs and malaria in the United States, merits attention and further exploration.

**Technical Appendix.** Diagnostic codes related to neurocysticercosis, region groups for states included in the Nationwide Inpatient Sample, and regression output for hospitalization charges and hospital stay.
