# Supplementary material for: Hospitalization Frequency and Charges for Neurocysticercosis, United States, 2003–2012
Source: Emerg Infect Dis. 2015 Jun;21(6):969–76. doi: 10.3201/eid2106.141324 (PMC4451927; doi:10.3201/eid2106.141324)
Supplement: Supplementary file 1 — Technical Appendix. Diagnostic codes related to neurocysticercosis, region groups for states included in the Nationwide Inpatient Sample, and regression output for hospitalization charges and hospital stay. [file 14-1324-Techapp-s1.pdf]

Article DOI: http://dx.doi.org/10.3201/eid2106.141324

Hospitalization Frequency and Charges for Neurocysticercosis, United States, 2003–2012

Technical Appendix

The Agency for Healthcare Research and Quality (AHRQ) developed the Clinical Classification Software (CCS) tool that groups International Classification of Diseases, 9th revision, Clinical Modification, (ICD-9-CM) diagnostic and procedural codes into clinically meaningful categories. Single-level CCS grouping codes were used in this study to identify hospitalizations for neurocysticercosis. The study case definition required an ICD-9-CM diagnostic code for cysticercosis (1231) *and* at least one other single-level CCS diagnostic or procedural code for a neurologic manifestation associated with neurocysticercosis.

CCS Diagnostic Codes

76 Meningitis (except that caused by tuberculosis or sexually transmitted disease)

00321 0360 0470 0471 0478 0479 0490 0491 0530 05472 0721 10081 11283 1142 11501 11511 11591 3200 3201 3202 3203 3207 3208 32081 32082 32089 3209 3210 3211 3212 3213 3214 3218 3220 3221 3222 3229

77 Encephalitis (except that caused by tuberculosis or sexually transmitted disease)

0361 0462 0498 0499 0520 0543 0550 05601 05821 05829 0620 0621 0622 0623 0624 0625 0628 0629 0630 0631 0632 0638 0639 064 0662 0722 1300 1390 3230 32301 32302 3231 3232 3234 32341 32342 3235 32351 32352 3236 32361 32362 32363 3237 32371 32372 3238 32381 32382 3239 34120 34121 34122

78 Other CNS infection and poliomyelitis

04500 04501 04502 04503 04510 04511 04512 04513 04520 04521 04522 04523 04590 04591 04592 04593 0460 0461 04611 04619 0463 04671 04672 04679 0468 0469 048 138 3240 3241 3249 326 V1202

83 Epilepsy; convulsions

3450 34500 34501 3451 34510 34511 3452 3453 3454 34540 34541 3455 34550 34551 3456 34560 34561 3457 34570 34571 3458 34580 34581 3459 34590 34591 7803 78031 78032 78033 78039

84 Headache; including migraine

33900 33901 33902 33903 33904 33905 33909 33910 33911 33912 33920 33921 33922 3393 33941 33942 33943 33944 33981 33982 33983 33984 33985 33989 3460 34600 34601 34602 34603 3461 34610 34611 34612 34613 3462 34620 34621 34622 34623 34630 34631 34632 34633 34640 34641 34642 34643 34650 34651 34652 34653 34670 34671 34672 34673 3468 34680 34681 34682 34683 3469 34690 34691 34692 34693 7840

85 Coma; stupor; and brain damage

3481 7800 78001 78003 78009

90 Inflammation; infection of eye (except that caused by tuberculosis or sexually transmitted disease)

0213 03281 05320 05321 05322 05329 05440 05441 05442 05443 05444 05449 05571 0760 0761 0769 0770 0771 0772 0773 0774 0778 0779 07798 07799 11502 11512 11592 1301 1302 1391 36000 36001 36002 36003 36004 36011 36012 36013 36014 36019 36300 36301 36303 36304 36305 36306 36307 36308 36310 36311 36312 36313 36314 36315 36320 36321 36322 36400 36401 36402 36403 36404 36405 36410 36411 36421 36422 36423 36424 3643 37020 37021 37022 37023 37024 37031 37032 37033 37034 37035 37040 37044 37049 37050 37052 37054 37055 37059 3708 3709 37200 37201 37202 37203 37204 37205 37206 37210 37211 37212 37213 37214 37215 37220 37221 37222 37230 37231 37233 37239 37300 37301 37302 37311 37312 37313 37331 37332 37333 37334 3734 3735 3736 3738 3739 37500 37501 37502 37503 37530 37531 37532 37533 37541 37542 37543 37600 37601 37602 37603 37604 37610 37611 37612 37613 37730 37731 37732 37733 37734 37739 37900 37901 37902 37903 37904 37905 37906 37907 37909 37960 37961 37962 37963

95 Other nervous system disorders

325 32702 32715 32730 32731 32732 32733 32734 32735 32736 32737 32739 32753 33183 3321 33720 33721 33722 33729 3380 33811 33812 33818 33819 33821 33822 33828 33829 3383 3384 3410 3411 3418 3419 34461 347 34700 34701 34710 34711 3480 3482 3483

34830 34831 34839 3484 3485 3488 34881 34882 34889 3489 3492 34981 34982 34989 3499 3501 3502 3508 3509 3510 3511 3518 3519 3520 3521 3522 3523 3524 3525 3526 3529 3530 3531 3532 3533 3534 3535 3536 3538 3539 3540 3541 3542 3543 3544 3545 3548 3549 3550 3551 3552 3553 3554 3555 3556 3557 35571 35579 3558 3559 3560 3561 3562 3563 3564 3568 3569 3570 3571 3572 3573 3574 3576 3577 3578 35781 35782 35789 3579 3580 35800 35801 3581 3582 35830 35831 35839 3588 3589 3590 3591 3592 35921 35922 35923 35924 35929 3593 3594 3595 3596 35971 35979 3598 35981 35989 3599 7810 7811 7812 7813 7817 7818 7820 7843 7845 78451 78452 78459 78460 78461 78469 7920 7930 79400 79401 79402 79409 79410 79411 79412 79413 79414 79415 79416 79417 79419 7961 79951 79952 79953 79954 79955 79959 V124 V1240 V1241 V1242 V1249 V415 V452 V484 V485 V493 V530 V5301 V5302 V5309

109 Acute cerebrovascular disease

34660 34661 34662 34663 430 431 4320 4321 4329 43301 43311 43321 43331 43381 43391 4340 43400 43401 4341 43410 43411 4349 43490 43491 436

111 Other and ill-defined cerebrovascular disease

4370 4371 4373 4374 4375 4376 4377 4378 4379

112 Transient cerebral ischemia

4350 4351 4352 4353 4358 4359

245 Syncope

7802

650 Adjustment disorders

3090 3091 30922 30923 30924 30928 30929 3093 3094 30982 30983 30989 3099

651 Anxiety disorders

29384 30000 30001 30002 30009 30010 30020 30021 30022 30023 30029 3003 3005 30089 3009 3080 3081 3082 3083 3084 3089 30981 3130 3131 31321 31322 3133 31382 31383

652 Attention-deficit, conduct, and disruptive behavior disorders

31200 31201 31202 31203 31210 31211 31212 31213 31220 31221 31222 31223 3124 3128 31281 31282 31289 3129 31381 31400 31401 3141 3142 3148 3149

653 Delirium, dementia, and amnestic and other cognitive disorders

2900 29010 29011 29012 29013 29020 29021 2903 29040 29041 29042 29043 2908 2909 2930 2931 2940 2941 29410 29411 29420 29421 2948 2949 3100 3102 3108 31081 31089 3109 3310 3311 33111 33119 3312 33182 797

656 Impulse control disorders, NEC

31230 31231 31232 31233 31234 31235 31239

657 Mood disorders

29383 29600 29601 29602 29603 29604 29605 29606 29610 29611 29612 29613 29614 29615 29616 29620 29621 29622 29623 29624 29625 29626 29630 29631 29632 29633 29634 29635 29636 29640 29641 29642 29643 29644 29645 29646 29650 29651 29652 29653 29654 29655 29656 29660 29661 29662 29663 29664 29665 29666 2967 29680 29681 29682 29689 29690 29699 3004 311

658 Personality disorders

3010 30110 30111 30112 30113 30120 30121 30122 3013 3014 30150 30151 30159 3016 3017 30181 30182 30183 30184 30189 3019

659 Schizophrenia and other psychotic disorders

29381 29382 29500 29501 29502 29503 29504 29505 29510 29511 29512 29513 29514 29515 29520 29521 29522 29523 29524 29525 29530 29531 29532 29533 29534 29535 29540 29541 29542 29543 29544 29545 29550 29551 29552 29553 29554 29555 29560 29561 29562 29563 29564 29565 29570 29571 29572 29573 29574 29575 29580 29581 29582 29583 29584 29585 29590 29591 29592 29593 29594 29595 2970 2971 2972 2973 2978 2979 2980 2981 2982 2983 2984 2988 2989

662 Suicide and intentional self-inflicted injury

E9500 E9501 E9502 E9503 E9504 E9505 E9506 E9507 E9508 E9509 E9510 E9511 E9518 E9520 E9521 E9528 E9529 E9530 E9531 E9538 E9539 E954 E9550 E9551 E9552 E9553 E9554 E9555 E9556 E9557 E9559 E956 E9570 E9571 E9572 E9579 E9580 E9581 E9582 E9583 E9584 E9585 E9586 E9587 E9588 E9589 E959 V6284

670 Miscellaneous mental disorders

29389 2939 30011 30012 30013 30014 30015 30016 30019 3006 3007 30081 30082 3021 3022 3023 3024 30250 30251 30252 30253 3026 30270 30271 30272 30273 30274 30275

30276 30279 30281 30282 30283 30284 30285 30289 3029 3060 3061 3062 3063 3064 30650 30651 30652 30653 30659 3066 3067 3068 3069 3071 30740 30741 30742 30743 30744 30745 30746 30747 30748 30749 30750 30751 30752 30753 30754 30759 30780 30781 30789 3101 316 64840 64841 64842 64843 64844 V402 V403 V4031 V4039 V409 V673

CCS Procedural Codes

One incision and excision of CNS

0101 0109 0121 0122 0123 0124 0125 0126 0127 0128 0131 0132 0139 0141 0142 0151 0152 0153 0159

Two insertion; replacement; or removal of extracranial ventricular shunt

0231 0232 0233 0234 0235 0239 0242 0243

177 Computerized axial tomography (CT) scan head

8703

198 Magnetic resonance imaging

0032 8891 8892 8893 8894 8895 8896 8897 8899

199 Electroencephalogram (EEG)

8914

Diagnostic Codes

(ICD-9-CM was used in this study to identify diagnostic codes for the neglected tropical diseases and malaria. The following diagnostic codes were used to evaluate the number of hospitalizations and total charges associated with the following diseases:

Buruli ulcer: (none)

Chagas: 0860 0861 0862 0869

Dengue: 061

Dracunculiasis: 1257

Echinococcus: 1220 1221 1222 1223 1224 1225 1226 1227 1228 1229

Foodborne trematodes: 1210 1211 1212 1213 1114 1215 1216 1218 1219

Human African trypanosomiasis: 0863 0864 0865 0869 3213

Leishmaniasis: 0850 0851 0852 0853 0854 0855 0859

Leprosy: 0300 0301 0302 0303 0308 0309

Lymphatic filariasis: 1250 1251 1256 1259

Malaria: 0840 0841 0842 0843 0844 0845 0846 0847 0848 0849

64740 64741 64742 64743 64744

Onchocerciasis: 1253

Rabies: 071

Schistosomiasis: 1200 1201 1202 1203 1208 1209

Soil transmitted helminthes: 1260 1261 1269 1270 1273

Taeniasis–cysticercosis: 1230 1231

Trachoma: 0760 0761 0769 1391

Yaws: 1020 1021 1022 1023 1024 1025 1026 1027 1028 1029

Technical Appendix Table 1. Region groups for states included in the Nationwide Inpatient Sample during years 2003–2012*

Region	States
Northeast	Connecticut, Maine, Massachusetts, New Hampshire, New Jersey, New York, Pennsylvania, Rhode Island, Vermont
Midwest	Illinois, Indiana, Iowa, Kansas, Michigan, Minnesota, Missouri, Nebraska, North Dakota, Ohio, South Dakota, Wisconsin
South	Arkansas, Florida, Georgia, Kentucky, Louisiana, Maryland, Mississippi, North Carolina, Oklahoma, South Carolina, Tennessee, Texas, Virginia, West Virginia
West	Alaska, Arizona, California, Colorado, Hawaii, Montana, Nevada, New Mexico, Oregon, Utah, Washington, Wyoming

*Not represented: Alabama, Delaware, District of Columbia, Idaho.

Technical Appendix Table 2. Regression output for univariate and multivariate generalized linear models with log link estimating mean hospitalization charges while controlling for year and demographic variables. Inflation-adjusted hospitalization charges are represented.*

		Univariate			Multivariate		
Variable		exp(b)	p >|t|	p >|F|†	exp(b)	p	95% CI
Year				<0.01			
	2003	Ref.	–		Ref.	–	–
	2004	0.95	0.7		0.99	1.0	0.76–1.29
	2005	0.96	0.8		1.06	0.7	0.82–1.37
	2006	1.17	0.3		1.19	0.3	0.88–1.62
	2007	1.30	0.1		1.27	0.2	0.87–1.87
	2008	1.38	0.1		1.62	0.1	0.94–2.81
	2009	1.10	0.5		1.04	0.8	0.79–1.35
	2010	1.32	0.04		1.35	0.1	0.98–1.86
	2011	1.56	<0.01		1.60	0.01	1.10–2.31
	2012	1.50	0.01		1.54	0.02	1.07–2.22

	Univariate			Multivariate		
Variable	exp(b)	p >|t|	p >|F|†	exp(b)	p	95% CI
Sex			<0.01			
M	Ref.	–		Ref.	–	–
F	0.85	<0.01		0.76	<0.01	0.64–0.91
Age			<0.01			
0–19	Ref.	–		Ref.	–	–
20–44	1.4	<0.01		1.23	0.3	0.84–1.79
45–64	1.8	<0.01		1.44	0.02	1.05–1.96
≥65	1.6	<0.01		1.14	0.5	0.74–1.77
Race			<0.01			
White	Ref.	–		Ref.	–	–
Black	0.93	0.7		1.42	0.4	0.68–3.00
Hispanic	1.05	0.6		1.08	0.6	0.81–1.43
Asian/Pacific Islander	0.74	0.1		0.99	1.0	0.64–1.52
Other	0.74	0.01		0.91	0.6	0.66–1.25
Region			<0.01			
Northeast	Ref.	–		Ref.	–	–
Midwest	0.70	<0.01		0.70	0.02	0.51–0.95
South	0.79	<0.01		0.82	0.02	0.69–0.97
West	1.44	<0.01		1.61	<0.01	1.18–2.19

*Ref., reference. †Adjusted Wald test.

Technical Appendix Table 3. Regression output for univariate and multivariate generalized linear models with log link estimating mean length of hospital stay while controlling for year and demographic variables.*

	Univariate			Multivariate		
Variable	exp(b)	p > |t|	p > |F|*	exp(b)	p	95% CI
Year			1.0			
2003	Ref.	–				
2004	1.04	0.7				
2005	1.00	1.0				
2006	1.08	0.5				
2007	1.08	0.5				
2008	1.08	0.5				
2009	1.04	0.8				
2010	1.08	0.6				
2011	1.13	0.3				
2012	1.07	0.6				
Sex			<0.01			
M	Ref.	–		Ref.	–	–
F	0.83	<0.01		0.82	<0.01	0.73–0.91
Age			<0.01			
0–19	Ref.	–		Ref.	–	–
20–44	1.31	<0.01		1.31	<0.01	1.12–1.53
45–64	1.46	<0.01		1.45	<0.01	1.20–1.74
≥65	1.34	<0.01		1.33	<0.01	1.10–1.62
Race			0.03			
White	Ref.	–		Ref.	–	–
Black	1.17	0.2		1.21	0.2	0.91–1.60
Hispanic	1.00	1.0		0.98	0.8	0.82–1.16
Asian/Pacific Island	0.79	0.1		0.80	0.1	0.62–1.04
Other	0.88	0.2		0.85	0.1	0.6–1.04
Region			<0.01			
Northeast	Ref.	–		Ref.	–	–
Midwest	0.73	<0.01		0.67	<0.01	0.56–0.79
South	0.88	0.06		0.87	0.06	0.76–1.01
West	0.96	0.5		0.97	0.7	0.83–1.12

*Ref., reference. †Adjusted Wald test.
